# Effects of coffee, smoking, and alcohol on liver function tests: a comprehensive cross-sectional study

**DOI:** 10.1186/1471-230X-12-145

**Published:** 2012-10-18

**Authors:** Eun Sun Jang, Sook-Hyang Jeong, Sung Ho Hwang, Hyun Young Kim, So Yeon Ahn, Jaebong Lee, Sang Hyub Lee, Young Soo Park, Jin Hyeok Hwang, Jin-Wook Kim, Nayoung Kim, Dong Ho Lee

**Affiliations:** 1 Department of Internal Medicine, Seoul National University Bundang Hospital, Seoul National University College of Medicine, 166 Gumi-ro, Bundang-gu, Seongnam-si, 463-707, Gyeonggi-do, Republic of Korea; 2 Health Promotion Center, Seoul National University Bundang Hospital, Seoul National University College of Medicine, 166 Gumi-ro, Bundang-gu, Seongnam-si, 463-707, Gyeonggi-do, Republic of Korea; 3 Medical Research Collaborating Center, Seoul National University Bundang Hospital, Seoul National University College of Medicine, 166 Gumi-ro, Bundang-gu, Seongnam-si, 463-707, Gyeonggi-do, Republic of Korea

**Keywords:** Coffee, Alcohols, Smoking, Liver function tests, Gamma-glutamyltransferase

## Abstract

**Background:**

Liver function tests (LFTs) can be affected by many factors and the proposed effects of coffee on LFT require a comprehensive evaluation. The aim of this study was to elucidate whether drinking coffee, smoking, or drinking alcohol have independent effects on LFTs in Korean health-check examinees.

**Methods:**

We used the responses of 500 health-check examinees, who had participated in a self-administered questionnaire survey about coffee, alcohol drinking, and smoking habits.

**Results:**

Coffee consumption was closely related to male gender, high body mass index (BMI), alcohol drinking, and smoking. On univariable and multivariable analyses, drinking coffee lowered serum levels of total protein, albumin, and aspartate aminotransferases (AST). On multivariable analyses, smoking raised serum γ-glutamyl transferase (GGT) level and decreased serum protein and albumin levels, while alcohol drinking raised GGT level after adjustment for age, gender, regular medication, BMI, coffee and alcohol drinking amounts, and smoking.

**Conclusions:**

Coffee consumption, smoking, and alcohol drinking affect the individual components of LFT in different ways, and the above 3 habits each have an impact on LFTs. Therefore, their effects on LFTs should be carefully interpreted, and further study on the mechanism of the effects is warranted.

## Background

Liver function tests (LFT) are useful tools in clinical practice to assess potential liver diseases, to monitor treatment responses, and to predict prognosis of the patients with liver diseases. As a battery, LFTs consists most commonly of serum total cholesterol (TC), total protein, albumin, alkaline phosphatase (ALP), total bilirubin (TB), aspartate amino transferase (AST), alanine aminotransferase (ALT), and γ-glutamyl transferase (GGT). However, the interpretation of LFTs should be comprehensive and careful because LFTs can be influenced by many personal and environmental factors, including age, gender [1], body mass index (BMI) [2], alcohol drinking [3], cigarette smoking, malnutrition, presence of extrahepatic diseases such as cardiac, musculoskeletal, or endocrine diseases, and status of liver health in itself [4].

Although the influence of alcohol drinking on the liver function has been extensively studied, studies on the effects of coffee drinking or cigarette smoking have been relatively limited. According to the international coffee organization, people drink approximately 2.25 billion cups of coffee everyday across the globe, and Korea is the 11^th^ highest coffee consuming country in the world, consuming 65 thousand tons of coffee a year. Considering the large amounts of consumed coffee globally and its lifetime consumption in most individuals, the effects of coffee on health merit great attention. Interestingly, coffee has been suggested to have a potential favorable impact on liver diseases. In North America and Europe, studies have shown that coffee drinking reduces the risk of liver cirrhosis and hepatocellular carcinoma (HCC) [5,6]. In addition, the protective effect of coffee drinking on the development of HCC has been reported in the Japanese [7]. Moreover, coffee consumption was inversely related with serum levels of ALT and GGT among LFT, especially in heavy alcohol drinkers [8].

Recently, it has been suggested that smoking increases GGT and boosts the alcohol-induced GGT elevations [9]. Although smoking does not damage hepatocytes directly, it may change the effect of alcohol drinking on AST, ALT and GGT activities via the actions of numerous ingredients that alter the activities of enzymes found in the liver [10]. Furthermore, smoking increased serum ALP levels [11] produced by the bone and the kidney, as well as by the liver. However, it is still not clear whether smoking is related to each component of LFTs and whether the effects are independent of alcohol drinking, since the smoking habit itself is closely related to the alcohol drinking habit [12]. Therefore, LFT changes in real clinical situations need to be interpreted carefully in the context of the interaction between various life style factors.

Nevertheless, most previous studies deal with the separate effects of alcohol, coffee, or smoking on one or a few components of LFTs, and there has been a lack of data on the comprehensive effects of alcohol, smoking and coffee drinking on the commonly used all components of LFTs. Moreover, those studies have been reported primarily from Western countries and Japan, while the study of the effects of these behaviors on LFTs for persons living in Asian countries have had limited study. Thus, this study was conducted in a Korean population to evaluate the effects of coffee consumption, smoking, and alcohol drinking on the most commonly used sets of LFTs in order to consider the interactive association with major demographic factors.

## Methods

### Patients

In this cross-sectional study, all health-check examinees were consecutively enrolled in the health promotion center of Seoul National University Bundang Hospital from August 2007 to October 2007. Of 909 examinees during this study period, 500 (55%) subjects agreed to participate to a comprehensive self-administered questionnaire survey about smoking, alcohol consumption, and coffee drinking habits. Moreover, they were requested to answer the questions about their current diseases undergoing medical care, their past histories of hypertension, of diabetes mellitus, of hypercholesterolemia, or of any other unspecified diseases and regular medications, in order to discern the effects of regular medication on serum LFTs. This study was approved by the Institutional Review Board of Seoul National University Bundang Hospital and all participants provided written informed consent prior to this study.

### Questionnaire

Detailed information on each subject’s demographics, medical history, physical findings, and laboratory results were obtained from medical records. The information on coffee and alcohol consumption, and cigarette smoking habits was collected from the self-reported questionnaire survey ( Additional file 1). Coffee consumers were classified as ‘none’, ‘past’, or ‘current drinker’. If a subject was a past or current drinker, information on total duration of coffee drinking and the daily amount of coffee consumed were requested. The types of the most consumed coffee were divided into 4 groups: instant, brewed, a combination of instant and brewed coffee, and others. The information about the duration of coffee consumption was obtained through an open question (years). The frequency and amount of coffee consumption was asked by closed-ended question with 9 options (No, < 1 cup/month, 2–3 cups/month, a cup/week, 2–3 cups/week, 4–6 cups/week, 1–2 cups/day, 3–4 cups/day, > 5 cups/day).

Alcohol drinking status was classified as none, past, or current drinker and information on the detailed alcohol drinking duration and daily amount of consumed alcoholic beverages were obtained with similar way to those of coffee consumption. Since an alcoholic consumption of more than 60 g/day in men (20 g/day in women) over more than 10 years has been documented as a major risk factor for alcoholic liver disease [13], we categorized alcohol drinkers according to their mean daily drinking amounts into none, not-heavy (<60 g/day in men, <20 g/day in women), and heavy (≥60 g/day in men, ≥20 g/day in women) drinkers, and into never, mild (<100 x 10^3^ g in men, <35 x 10^3^ g in women), moderate (100–200 x 10^3^ g in men, 35–70 x 10^3^ g in women), and heavy lifetime drinkers (≥ 200 x 10^3^ g in men, ≥70 x 10^3^ g in women), according to their lifetime drinking amounts which were calculated based on the number of years that a person had been drinking and the daily amounts of alcohol consumption.

Smoking histories were classified in a similar manner as above, participants were asked about their mean cigarette amounts (packs) smoked per day over the previous 6 months and the number of years having smoked. Then, participants were classified into none, mild (<0.5 pack/day), moderate (0.5-1 pack/day), and heavy daily smokers (> 1 packs/day) and into never, mild (<10 pack-years), moderate (10–20 pack-years), and heavy (≥20 pack-years) lifetime smokers.

### Physical and biochemical measurements

The height and weight of each participant were measured in order to calculate BMI. Biochemical assays for serum TC, total protein, albumin, ALP, TB, AST, ALT and GGT were performed in all subjects. The serologic status of chronic viral hepatitis was evaluated by assays for hepatitis B surface antigen (HBsAg)/antibody (HBsAb) and anti-hepatitis C virus antibody (anti-HCV). All assays were performed at the laboratory of Seoul National University Bundang Hospital, which complied with the International Federation of Clinical Chemistry and Laboratory Medicine. We performed abdominal sonography in all subjects, which was included in the routine health-check program of our hospital.

### Statistical analysis

The LFTs were recorded as continuous variables, but serum GGT level was transformed to a natural logarithmic scale to normalize its non-standard distribution. To compare baseline characteristics of subgroups, univariable analyses were performed using Student T tests or analysis of variance (ANOVA) for continuous variables and χ^2^ or tests for trend using linear-by linear association for categorized variables.

Estimated means and 95% confidence intervals(CI) for each LFT result were calculated from multivariable linear regression models after adjustment for age, gender, BMI, regular medication for hypertension/diabetes mellitus/hypercholesterolemia, daily alcohol drinking (none, male (M) < 60 g or female (F) < 20 g, M ≥ 60 g or F ≥ 20 g), and smoking (none, < 0.5 pack, 0.5-1 pack, >1 packs) histories to determine the association between coffee drinking and each LFT test result. To estimate independent effects of alcohol or smoking on LFTs, the same multivariable analyses were performed after adjustment for daily coffee consumption amounts (none, < 1 cup, 1–2 cups, ≥ 3 cups), and smoking or alcohol drinking amounts, separately.

All statistical analyses were performed using SPSS 17.0 (SPSS Institute, Inc.; Chicago, IL, USA) software. A *P*-value of <0.05 was considered statistically significant on all analyses.

## Results

### Baseline characteristics of participants

The mean age of all participants (n=500) was 46.5 years. Mean BMI was 23.5 kg/m^2^ and 144 (28.8%) were obese (BMI ≥ 25 kg/m^2^) [14]. HBsAg was positive in 19 (3.8%) subjects and anti-HCV was detected in 1 (0.2%) subjects, however, viral loads of those subjects were not detectable. Fatty liver on the ultrasonography was detected in 46 (9.2%) participants.

A total of 443 (88.6%) participants were coffee drinkers at the time of the study and only 38 (7.6%) were never-coffee drinkers (Table 1), and more than half of Korean coffee drinkers preferred instant coffee (57.8%) to brewed (8.0%, Table 1). The comparison of demographics, alcohol drinking, and smoking patterns among coffee consumer groups categorized by daily coffee amounts was summarized in Table 2. Interestingly, coffee consumption was closely related to alcohol drinking and smoking habits. As shown in Table 2, heavy coffee consumers were more often current alcohol drinkers, and more often heavy daily smokers. The interaction was more consistent between coffee consumption and smoking than between coffee consumption and alcohol drinking.

**Table 1:** Baseline characteristics of study population

	**Total**	**Male**	**Female**	*p*-value^*^
	**N=500**	**n=268**	**n=232**	
**Age**^a^, years	46.5 (10.8)	46.7 (10.8)	46.2 (10.8)	0.432
**BMI**^a^, kg/m^2^	23.5 (3.4)	24.5 (3.5)	22.3 (2.9)	<0.001
Obesity (BMI≥25)^b^	144 (28.8)	107 (39.9)	37 (15.9)	<0.001
Overweight (BMI ≥23)^b^	269 (53.8)	190 (70.9)	79 (34.1)	<0.001
**Regular medication**^b^	83 (16.6)	50 (18.7)	33 (14.2)	0.188
Anti-hypertensive drug	69 (13.8)	42 (15.7)	27 (11.7)	0.242
Oral hypoglycemic agent	9 (1.8)	7 (2.6)	2 (0.9)	0.186
Anti-hypercholesterolemic drug	13 (2.6)	7 (2.6)	6 (2.6)	1.000
**Ultrasonography**^b^				
Fatty liver	46 (9.2)	37 (13.8)	9 (3.9)	<0.001
**Liver function tests**^a^				
Total cholesterol, mg/dL	198.4 (35.2)	201.6 (33.9)	194.9 (36.3)	0.030
Total protein, g/dL	7.11 (0.37)	7.11 (0.36)	7.11 (0.39)	0.970
Albumin, g/dL	4.44 (0.24)	4.50 (0.24)	3.38 (0.22)	<0.001
Total bilirubin, mg/dL	1.07 (0.41)	1.17 (0.38)	0.96 (0.41)	<0.001
ALP, IU/L	63.2 (18.6)	65.3 (15.9)	60.8 (21.1)	0.008
AST, IU/L	24.3 (12.4)	25.8 (11.8)	22.6 (12.8)	0.005
ALT, IU/L	25.8 (18.0)	30.7 (18.0)	20.1 (16.3)	<0.001
GGT, IU/L	35.3 (39.4)	46.0 (35.7)	33.9 (39.9)	<0.001
Ln(GGT)	3.25 (0.72)	3.60 (0.64)	2.83 (0.58)	<0.001
**Coffee**				
**Drinking history**^b^				
None drinker	38 (7.6)	19 (7.1)	19 (8.2)	0.525^†^
Past drinker, quitted	19 (3.8)	9 (3.4)	10 (4.3)	
Current drinker	443 (88.6)	240 (89.6)	203 (87.5)	
**Type of coffee**^b^				
Instant	289 (57.8)	164 (61.2)	125 (53.9)	0.084^†^
Brewed	40 (8.0)	21 (7.8)	19 (8.2)	
Instant and brewed (mixed)	66 (13.2)	33 (12.3)	33 (14.2)	
Others	105 (21.0)	50 (18.7)	55 (23.7)	
**Daily amount of coffee consumption (cups/day)**^b^				
0	43 (8.6)	22 (8.2)	21 (9.1)	0.001^†^
<1	109 (21.8)	51 (19.0)	58 (25.0)	
1-2	229 (45.6)	108 (40.3)	120 (51.7)	
≥ 3	120 (24.0)	87 (32.5)	33 (14.2)	
**Smoking**				
**History**^b^				
None smoker	262 (53.3)	50 (18.7)	212 (94.2)	<0.001^†^
Past smoker, quitted	111 (22.6)	109 (40.8)	2 (0.9)	
Current smoker	119 (24.2)	108 (40.4)	11 (4.9)	
**Daily smoking amount (packs/day)**^b^	n=473	n=249	n=224	
No	262 (55.4)	50 (20.1)	212 (94.6)	<0.001^†^
<0.5	76 (16.1)	66 (26.5)	10 (4.5)	
0.5-1	96 (20.3)	94 (37.8)	2 (0.9)	
> 1	39 (8.2)	39 (15.7)	0 (0.0)	
**Alcohol**				
**Drinking history**^b^				
None drinker	173 (34.6)	32 (11.9)	141 (60.8)	<0.001^†^
Past drinker, quitted	19 (3.8)	13 (4.9)	6 (2.6)	
Current drinker	308 (61.6)	223 (83.2)	85 (36.6)	
**Daily amount of alcohol consumption**^b^				
No	180 (36.0)	34 (12.7)	146 (62.9)	<0.001^†^
Not heavy (< 60g/day for male, <20g/day for female)	290 (58.0)	213 (79.5)	77 (33.2)	
Heavy (≥ 60 g/day for male, ≥20 g/day for female)	30 (6.0)	21 (7.8)	9 (3.9)	
**Duration of drinking**^b^				
≥ 10 years	236 (49.4)	203 (76.0)	43 (18.6)	<0.001

Abbreviation:BMI, body mass index; ALP, alkaline phosphatase; AST, aspartate aminotransferase; ALT, alanine aminotransferase; Ln(GGT), natural logarithmic scale of gamma-glutamyltransferase.

^a^ Data are presented as mean (SD).

^b^ Data are presented as number (%).

^*^ P-value for the comparison between men and women.

^†^ P for trend.

**Table 2:** Comparison of characteristics among coffee consumer groups categorized by daily coffee amounts

	**Daily coffee drinking amount**				
	**No**	**Mild**	**Moderate**	**Heavy**	
	**0 cup/day**	**<1 cup/day**	**1-2 cup/day**	**≥3 cup/day**	
	**n=43**	**n=109**	**n=228**	**n=120**	***p*-value**
**Age**^a^, years	48.5 (12.7)	44.6 (12.3)	47.4 (9.7)	45.7 (10.3)	0.077
**Male**^b^	22 (51.2)	51 (46.8)	108 (47.4)	87 (72.5)	0.001
**BMI**^a^	23.2 (2.8)	23.4 (3.1)	23.1 (3.1)	24.2 (4.0)	0.035
Obesity (BMI≥25 kg/m^2^)^b^	11 (25.6)	26 (23.9)	63 (27.6)	44 (36.7)	0.152
**Regular medication**^b^	8 (18.6)	20 (18.3)	39 (17.1)	16 (13.3)	0.305
Anti-hypertensive drug	7 (16.3)	19 (17.4)	29 (12.8)	14 (11.7)	0.206
Oral hypoglycemic agent	2 (4.7)	1 (0.9)	5 (2.2)	1 (0.8)	0.313
Anti-dyslipidemic drug	1 (2.3)	4 (3.7)	7 (3.1)	1 (0.8)	0.332
**Ultrasonography**					
Fatty liver^b^	2 (4.7)	13 (11.9)	17 (7.5)	14 (11.8)	0.480
**Alcohol**					
**Drinking history**^b^					
None drinker	22 (51.2)	37 (33.9)	81 (35.5)	33 (27.5)	0.007^†^
Past drinker, quitted	3 (7.0)	7 (6.4)	5 (2.2)	4 (3.3)	
Current drinker	18 (41.9)	65 (59.6)	142 (62.3)	83 (69.2)	
**Daily amount of alcohol consumption**^b^	15.9 (40.9)	11.1 (19.9)	14.0 (34.9)	16.0 (27.1)	0.649
No	22 (51.2)	40 (36.7)	83 (36.4)	35 (29.2)	0.086^†^
Not heavy (< 60g/day for male, <20g/day for female)	17 (39.5)	63 (57.8)	131 (57.5)	79 (65.8)	
Heavy (≥ 60 g/day for male, ≥20 g/day for female)	4 (9.3)	6 (5.5)	14 (6.1)	6 (5.0)	
**Duration of drinking**^b^	n=43	n=106	n=227	n=116	
≥ 10 years	17 (39.5)	41 (38.7)	119 (52.4)	71 (61.2)	0.001
**Lifetime amount (x10^3^ g)**^b^	n=43	n=106	n=227	n=118	
Never	22 (51.2)	38 (35.8)	80 (35.2)	33 (28.0)	0.033^†^
Mild(<100 x10^3^ g for male , <35 x10^3^ g for female)	12 (27.9)	44 (41.5)	82 (36.1)	47 (39.8)	
Moderate (100–200 x10^3^ g for male, 35–70 x10^3^ g for female)	1 (2.3)	7 (6.6)	19 (8.4)	13 (11.0)	
Heavy (≥200 x10^3^ g for male, ≥70 x10^3^ g for female)	8 (18.6)	17 (16.0)	46 (20.3)	25 (21.2)	
**Smoking**					
**History**^b^	n=42	n=107	n=223	n=120	
None smoker	28 (66.7)	72 (67.3)	131 (58.7)	31 (25.8)	<0.001^†^
Past smoker, quitted	10 (23.8)	16 (15.0)	53 (23.8)	32 (26.7)	
Current smoker	4 (9.5)	19 (17.8)	39 (17.5)	57 (47.5)	
**Daily smoking amount (packs/day)**^b^	n=42	n=105	n=213	n=113	
No	28 (66.7)	72 (68.6)	131 (61.5)	31 (27.4)	<0.001^†^
<0.5	7 (16.7)	13 (12.4)	33 (15.5)	23 (20.4)	
0.5-1	4 (9.5)	16 (15.2)	34 (16.0)	42 (37.2)	
> 1	3 (7.1)	4 (3.8)	15 (7.0)	17 (15.0)	
**Lifetime amount (pack-year)**^b^	n=39	n=96	n=199	n=99	
Never	28 (71.8)	72 (75.0)	131 (65.8)	31 (31.3)	<0.001^†^
<10	5 (12.8)	12 (12.5)	27 (13.6)	15 (15.2)	
10-20	1 (2.6)	3 (3.1)	23 (11.6)	30 (30.3)	
≥ 20	5 (12.8)	9 (9.4)	18 (9.0)	23 (23.2)	

Abbreviation: BMI, body mass index.

^a^ Data are presented as mean (SD).

^b^ Data are presented as number (%).

^*^ P-value for the comparison among the daily coffee consumer groups (no, <1 cups/day, 1–2 cups/day, ≥3 cups/day).

^†^ P for trend.

### The association between coffee consumption and LFTs

The results of univariable analyses on the association between daily amount of coffee consumption, smoking, or alcohol drinking and each individual test of LFT are summarized in Table 3.

**Table 3:** Effects of coffee, smoking and alcohol on liver function tests by univariate analyses^a^

		**N**	**Total cholesterol, g/dL**	**Total protein, g/dL**	**Albumin, g/dL**	**Alkaline phosphatase, IU/L**	**AST, IU/L**	**ALT, IU/L**	**Ln(GGT)**	**Total bilirubin, mg/dL**
**Daily coffee drinking amount (cups/day)**	**No**	43	197.5 (38.9)	7.29 (0.36)	4.55 (0.25)	70.2 (23.0)	24.6 (7.8)	25.4 (13.1)	3.22 (0.68)	1.05 (0.40)
	**Mild (<1)**	109	194.4 (30.9)	7.13 (0.35)	4.44 (0.26)	62.5 (15.2)	27.4 (20.7)	28.2 (25.9)	3.18 (0.78)	1.09 (0.43)
	**Moderate (1–2)**	228	198.2 (36.9)	7.10 (0.39)	4.43 (0.22)	61.9 (19.6)	23.1 (8.9)	23.8 (14.8)	3.20 (0.68)	1.08 (0.42)
	**Heavy (≥3)**	120	202.8 (33.9)	7.04 (0.35)	4.44 (0.23)	63.7 (17.3)	23.7 (8.3)	27.6 (15.9)	3.40 (0.75)	1.06 (0.37)
	*p*-value		*0.344*	*0.002*	*0.025*	*0.057*	*0.029*	*0.111*	*0.051*	*0.888*
**Daily smoking amount (pack/day)**	**No**	262	196.2 (36.3)	7.16 (0.39)	4.43 (0.23)	61.4 (20.0)	22.3 (7.5)	21.3 (14.3)	2.93 (0.61)	1.00 (0.39)
	**Mild (<0.5)**	76	200.1 (32.4)	7.07 (0.37)	4.48 (0.35)	62.8 (15.3)	27.3 (22.7)	30.2 (22.8)	3.51 (0.76)	1.18 (0.45)
	**Moderate (0.5-1)**	96	200.1 (34.7)	7.03 (0.35)	4.46 (0.24)	66.6 (17.1)	25.5 (10.0)	31.8 (20.3)	3.63 (0.65)	1.15 (0.39)
	**Heavy (>1)**	39	203.5 (30.5)	7.04 (0.34)	4.43 (0.20)	64.0 (15.6)	25.8 (9.4)	30.8 (17.9)	3.82 (0.61)	1.09 (0.32)
	*p*-value		0.524	0.01	0.344	0.124	0.003	<0.001	<0.001	<0.001
**Daily drinking amount (g/day)**	No	180	200.5 (34.1)	7.14 (0.35)	4.41 (0.22)	64.9 (21.4)	24.3 (14.3)	23.0 (17.3)	2.93 (0.57)	0.97 (0.36)
	Not heavy (< 60g/day for male, <20g/day for female)	290	196.7 (35.5)	7.09 (0.39)	4.47 (0.24)	61.9 (15.7)	23.7 (8.2)	26.5 (16.7)	3.36 (0.68)	1.13 (0.42)
	Heavy ( ≥ 60 g/day for male, ≥20 g/day for female)	30	203.2 (37.7)	7.12 (0.38)	4.43 (0.24)	65.2 (25.3)	30.5 (25.7)	35.6 (28.1)	4.08 (0.94)	1.17 (0.45)
	*p*-value		0.394	0.369	0.03	0.198	0.016	0.001	<0.001	<0.001

Abbreviation:AST, aspartate aminotransferase; ALT, alanine aminotransferase; Ln(GGT), natural logarithmic scale of gamma-glutamyltransferase.

^a^ Data are presented as mean (SD).

Among the tests, serum total protein, albumin and AST levels were significantly affected by coffee consumption according to univariable analysis. Then, we performed multivariable analyses on the association between the coffee consumption and LFT changes. The results are summarized in Table 4, showing that current coffee drinkers had significantly lower serum total protein, albumin and ALP levels compared with never or past coffee drinkers. Among the current coffee consumers, heavy daily coffee drinkers (≥3 cups/day) showed significantly lower levels of serum total protein, albumin and AST levels. Moreover, heavy lifetime coffee drinkers (>20,000 cups/lifetime) showed significantly lower levels of TC, total protein, total albumin and AST, while ALT or GGT was not associated independently with coffee consumption history or total amounts after adjustments.

**Table 4:** The effects of coffee consumption on the liver function tests by multivariable analyses^a^

	**n**	**Total cholesterol, g/dL**	**Total protein, g/dL**	**Albumin, g/dL**	**Alkaline phosphatase, IU/L**	**AST, IU/L**	**ALT, IU/L**	**Ln(GGT)**	**Total bilirubin, mg/dL**
**1) History of coffee**									
None drinker	37	197.0 (184.4, 209.6)	7.24 (7.11, 7.48)	4.52 (4.44, 4.60)	68.7 (62.0, 75.1)	26.6 (22.2, 30.9)	28.8 (22.7, 35.0)	3.51 (3.30, 3.72)	1.01 (0.87, 1.15)
Past drinker, quitted	19	189.8 (173.3, 206.4)	7.20 (7.02, 7.37)	4.52 (4.42, 4.63)	72.7 (64.1, 81.3)	25.8 (20.1, 31.4)	27.5 (19.4, 35.6)	3.47 (3.19, 3.75)	0.99 (0.80, 1.18)
Current drinker	436	198.9 (192.2, 205.6)	7.07 (7.00, 7.14) *	4.42 (4.38, 4.46) *	63.9 (60.4, 67.4)	26.6 (24.4, 28.9)	29.0 (25.7, 32.2)	3.52 (3.40, 3.63)	1.08 (1.00, 1.15)
*p for trend*	492	*0.528*	*0.003*	*0.003*	*0.042*	*0.898*	*0.879*	*0.859*	*0.221*
**2) Daily amount of coffee consumption (cups/day)**									
No	42	195.7 (183.7, 207.7)	7.26 (7.13, 7.38)	4.53 (4.45, 4.61)	70.5 (64.2, 76.7)	26.5 (22.5, 30.5)	28.9 (23.1, 34.7)	3.51 (3.31, 3.71)	1.02 (0.88, 1.15)
<1	107	196.4 (187.5, 205.4)	7.10 (7.00, 7.20) *	4.43 (4.37, 4.48) *	65.5 (60.8, 70.2)	31.3 (28.3, 34.3)*	33.2 (28.8, 37.5)	3.54 (3.39, 3.69)	1.11 (1.01, 1.21)
1-2	223	197.8 (190.3, 205.3)	7.09 (8.01, 7.17) *	4.43 (4.38, 4.48) *	63.7 (59.8, 67.6) *	25.6 (23.1, 28.1)	27.8 (24.2, 31.4)	3.52 (3.40, 3.65)	1.09 (1.00, 1.18)
≥3	120	200.9 (192.4, 209.4)	7.04 (6.95, 7.13) *	4.42 (4.37, 4.48) *	64.1 (59.7, 68.5) *	25.1 (22.2, 27.9)	27.7 (23.6, 31.9)	3.49 (3.35, 3.63)	1.03 (0.93, 1.13)
*p for trend*	492	*0.326*	*0.005*	*0.048*	*0.068*	*0.006*	*0.088*	*0.733*	*0.691*
**3) Duration of coffee drinking (years)**									
Never	37	196.6 (184.0, 209.1)	7.25 (7.11, 7.38)	4.52 (4.44, 4.60)	69.1 (62.5, 75.6)	26.6 (22.3, 30.9)	28.7 (22.5, 34.8)	3.50 (3.30, 3.71)	1.00 (0.87, 1.15)
<10	93	191.9 (182.5, 201.3)	7.06 (6.96, 7.17) *	4.45 (4.39, 4.51)	68.2 (63.3, 73.1)	27.9 (24.7, 31.1)	28.6 (23.9, 33.2)	3.43 (3.29, 3.59)	1.08 (0.97, 1.19)
11-29	299	198.9 (192.0, 205.9)	7.08 (7.00, 7.15) *	4.43 (4.38, 4.47) *	63.3 (59.6, 67.0)	26.6 (24.3, 29.0)	29.7 (26.3, 33.0)	3.55 (3.43, 3.66)	1.07 (0.99, 1.15)
≥30	59	203.0 (191.6, 214.3)	7.11 (6.99, 7.24)	4.41 (4.33, 4.48) *	65.3 (59.4, 71.2)	24.6 (20.7, 28.5)	25.1 (19.6, 30.7)	3.45 (3.26, 3.64)	1.10 (0.97, 1.23)
*p for trend*	488	*0.132*	*0.144*	*0.013*	*0.046*	*0.295*	*0.633*	*0.642*	*0.396*
**4) Lifetime amount of coffee consumption (cups)**									
0	42	195.0 (183.1, 207.0)	7.25 (7.13, 7.38)	4.53 (4.45, 4.61)	70.4 (64.2, 76.6)	26.6 (22.5, 30.6)	28.9 (23.1, 34.8)	3.51 (3.30, 3.71)	1.02 (0.88, 1.15)
1-10000	233	194.2 (186.5, 201.9)	7.12 (7.04, 7.20) *	4.44 (4.39, 4.49) *	64.8 (60.8, 68.9)	29.0 (26.4, 31.6)	30.7 (27.0, 34.5)	3.53 (3.40, 3.66)	1.12 (1.03, 1.21)
10001-20000	139	198.5 (190.5, 206.5)	7.02 (6.93, 7.10) *	4.40 (4.35, 4.46) *	62.8 (58.6, 67.0) *	25.2 (22.5, 28.0)	27.9 (23.9, 31.8)	3.50 (3.37, 3.63)	1.04 (0.95, 1.13)
>20000	78	207.3 (196.5, 215.9)	7.08 (6.98, 7.19) *	4.44 (4.38, 4.50) *	65.2 (60.1, 70.3)	24.4 (21.2, 27.7)	27.2 (22.4, 31.9)	3.50 (3.34, 3.66)	1.05 (0.94, 1.16)
*p for trend*	492	*0.029*	*0.005*	*0.046*	*0.155*	*0.015*	*0.18*	*0.737*	*0.428*

Abbreviation:AST, aspartate aminotransferase; ALT, alanine aminotransferase; Ln(GGT), natural logarithmic scale of gamma-glutamyltransferase.

^a^ Data are presented as mean (95% C.I.). Estimated mean and 95% CI were calculated by using multivariable linear regression model adjusted for age, gender, body mass index, regular medication, daily alcohol drinking (none, M < 60 g or F < 20 g, M ≥ 60 g or F ≥ 20 g) and smoking (none, < 0.5 pack, 0.5-1 pack, > 1 packs) amounts.

*P-value <0.05 compared to none or never drinkers.

### The association between smoking and LFTs

Of 500 participants, 119 (24.2%) were current smokers (Table 1). In univariable analyses, the serum levels of total protein, AST, ALT, GGT, and TB were significantly affected by daily smoking amounts. Total protein level decreased as daily smoking amounts increased, while AST, ALT, and GGT levels increased as daily smoking amount increased (Table 3).

In the multivariable models adjusted for age, gender, BMI, regular medication, daily amounts of alcohol and coffee drinking, current smokers had significantly lower serum levels of total protein, and albumin, but higher GGT levels compared with never or past smokers (Table 5). In addition, daily and lifetime smoking amounts significantly affected serum total protein, albumin, and GGT levels, but did not affect AST or ALT (Table 5).

**Table 5:** The effects of smoking on the liver function tests by multivariable analyses^a^

	**n**	**Total cholesterol, g/dL**	**Total protein, g/dL**	**Albumin, g/dL**	**Alkaline phosphatase, IU/L**	**AST, IU/L**	**ALT, IU/L**	**Ln(GGT)**	**Total bilirubin, mg/dL**
**1) History of smoking**									
None smoker	262	199.3 (192.1, 206.6)	7.25 (7.18, 7.33)	4.52 (4.47, 4.56)	65.2 (61.4, 69.0)	24.1 (21.7, 26.5)	26.0 (22.6, 29.5)	3.35 (3.23, 3.48)	1.07 (0.99, 1.15)
Past smoker, quitted	111	196.0 (186.6, 205.4)	7.17 (7.07, 7.27)	4.47 (4.41, 4.53)	63.3 (58.4, 68.2)	28.7 (25.6, 31.8) *	31.4 (27.0, 35.9) *	3.50 (3.35, 3.66)	1.15 (1.05, 1.26)
Current smoker	119	200.6 (191.4, 209.9)	7.02 (6.92, 7.11) *	4.41 (4.35, 4.46) *	69.8 (65.0, 74.6)	27.9 (24.9, 30.9) *	30.0 (35.6, 34.4)	3.58 (3.43, 3.73) *	1.02 (0.91, 1.12)
*p for trend*	492	*0.672*	*<0.001*	*0.001*	*0.04*	*0.074*	*0.213*	*0.014*	*0.185*
**2) Daily amount of smoking (pack/day)**									
No	262	198.7 (191.3, 206.0)	7.26 (7.19, 7.34)	4.53 (4.48, 4.57)	64.9 (61.1, 68.7)	24.0 (21.6, 26.6)	26.0 (22.4, 29.5)	3.34 (3.22, 3.46)	1.07 (0.99, 1.16)
<0.5	76	197.8 (187.8, 207.8)	7.11 (7.00, 7.21) *	4.46 (4.40, 4.53)	65.0 (59.8, 70.3)	29.6 (26.3, 33.0) *	31.7 (26.9, 36.6) *	3.53 (3.36, 3.69)	1.11 (1.00, 1.22)
0.5-1	96	196.9 (186.9, 206.9)	7.06 (6.95, 7.17) *	4.42 (4.35, 4.48) *	69.1 (63.9, 74.3)	27.7 (24.3, 31.0)	31.3 (26.4, 36.1)	3.55 (3.39, 3.72) *	1.06 (0.95, 1.18)
> 1	39	197.5 (184.8, 210.1)	7.05 (6.92, 7.18) *	4.40 (4.32, 4.48) *	64.8 (58.1, 71.4)	27.1 (22.9, 31.3)	28.6 (22.5, 34.8)	3.64 (3.42, 3.85) *	0.99 (0.85, 1.14)
*p for trend*	473	*0.806*	*0.002*	*0.001*	*0.502*	*0.265*	*0.319*	*0.013*	*0.327*
3) Lifetime amount of smoking (pack*year)									
Never	262	198.0 (190.5, 205.6)	7.27 (7.19, 7.35)	4.53 (4.48, 4.58)	65.2 (61.2, 69.1)	24.9 (22.7, 27.1)	26.2 (22.7, 29.7)	3.35 (3.22, 3.47)	1.07 (0.99, 1.16)
< 10	59	194.7 (183.9, 205.5)	7.08 (6.96, 7.19) *	4.44 (4.37, 4.51) *	68.3 (62.7, 74.0)	28.5 (25.4, 31.6) *	30.7 (25.7, 35.7)	3.52 (3.34, 3.70)	1.10 (0.98, 1.22)
10 - 20	57	199.7 (187.8, 211.6)	7.08 (6.95, 7.21) *	4.45 (4.37, 4.53)	71.6 (65.4, 77.7)	27.6 (24.2, 31.0)	28.4 (22.8, 33.9)	3.56 (3.36, 3.75)	1.12 (0.98, 1.25)
≥ 20	55	198.9 (187.2, 210.6)	6.99 (6.97, 7.12) *	4.38 (4.31, 3.36) *	66.1 (60.0, 72.2)	28.0 (24.7, 31.4)	33.1 (27.7, 38.6) *	3.59 (3.40, 3.78) *	0.95 (0.82, 1.08)
*p for trend*	433	*0.755*	*<0.001*	*0.002*	*0.611*	*0.144*	*0.051*	*0.029*	*0.153*

Abbreviation:AST, aspartate aminotransferase; ALT, alanine aminotransferase; Ln(GGT), natural logarithmic scale of gamma-glutamyltransferase.

^a^ Data are presented as mean (95% C.I.). Estimated mean and 95% CI were calculated by using multivariable linear regression model adjusted for age, gender, body mass index, regular medication, daily alcohol (none, M < 60 g or F < 20 g, M ≥ 60 g or F ≥ 20 g) and coffee (none, < 1 cup, 1–2 cups, ≥ 3 cups) drinking amounts.

*P-value <0.05 compared to none or never drinkers.

### The association between alcohol and LFTs

Of our study population, 173 (34.6%) did not drink alcoholic beverages, 19 (3.8%) had quit drinking, and 308 (61.6%) were current alcohol drinkers (Table 1). In univariable analyses, heavy daily alcohol drinking was associated with lower levels of serum albumin and high levels serum AST, ALT, GGT and TB levels (Table 3). However, the increases in AST and ALT levels as the result of daily alcohol consumption did not maintain statistical significance after multivariable adjustments for age, gender, BMI, regular medication, coffee drinking, and smoking (Table 6), which showed that primarily ALP and GGT were affected by alcohol drinking. As shown in Table 6, mean adjusted ALP levels were affected by current alcohol drinking and heavy lifetime drinking amounts; however, these effects did not demonstrate a consistent tendency or pattern. GGT levels were undoubtedly increased in current drinkers and in heavy daily drinkers, as well as in heavy lifetime alcohol drinkers (Table 6).

**Table 6:** The effects of alcohol drinking on the liver function tests by multivariable analyses^a^

	**n**	**Total cholesterol, g/dL**	**Total protein, g/dL**	**Albumin, g/dL**	**Alkaline phosphatase, IU/L**	**AST, IU/L**	**ALT, IU/L**	**Ln(GGT)**	**Total bilirubin, mg/dL**
**1) History of alcohol drinking**									
None drinker	173	200.2 (192.0, 208.3)	7.13 (7.04, 7.21)	4.46 (4.41, 4.52)	68.6 (64.3, 72.9)	25.8 (23.0, 28.5)	28.7 (24.8, 32.7)	3.27 (3.13, 3.41)	1.00 (0.91, 1.10)
Past drinker, quit	19	189.2 (172.2, 206.4)	7.13 (6.94, 7.31)	4.49 (4.39, 4.60)	58.0 (49.2, 66.9) *	29.3 (23.5, 35.1)	28.6 (20.3, 37.0)	3.10 (2.81, 3.40)	1.00 (0.80, 1.19)
Current drinker	308	195.5 (188.7, 202.3)	7.08 (7.01, 7.16)	4.43 (4.39, 4.48)	64.0 (60.4, 67.5) *	25.0 (22.8, 27.3)	27.3 (24.0, 30.6)	3.43 (3.31, 3.55) *	1.07 (0.99, 1.14)
*p for trend*	500	*0.269*	*0.291*	*0.225*	*0.037*	*0.514*	*0.441*	*0.016*	*0.156*
**2) Daily amount of alcohol (g)**									
No	180	199.8 (191.7, 207.9)	7.13 (7.05, 7.22)	4.46 (4.41, 4.52)	68.2 (63.9, 72.4)	26.7 (24.0, 29.5)	29.1 (35.1, 33.0)	3.23 (3.10, 3.37)	0.99 (0.89, 1.08)
Not heavy (< 60g/day for male, <20g/day for female)	290	194.1 (187.3, 200.9)	7.07 (7.00, 7.15)	4.44 (4.40, 4.48)	62.8 (59.3, 66.3) *	24.2 (21.9, 26.5)	26.3 (23.0, 29.6)	3.31 (3.20, 3.43)	1.06 (0.98, 1.13)
Heavy (≥ 60 g/day for male, ≥20 g/day for female)	30	199.2 (185.8, 212.5)	7.15 (7.01, 7.30)	4.45 (4.37, 4.54)	66.8 (59.9, 73.8)	30.4 (25.9, 34.9)	32.8 (26.3, 39.3)	4.00 (3.77, 4.22) *	1.14 (0.99, 1.29)
*p for trend*	500	*0.412*	*0.593*	*0.471*	*0.111*	*0.963*	*0.939*	*<0.001*	*0.041*
**3) Duration of alcohol drinking (years)**									
<10	258	195.7 (188.1, 203.3)	7.12 (7.04, 7.20)	4.46 (4.41, 4.51)	67.1 (36.1, 71.1)	25.6 (23.0, 28.1)	26.7 (23.0, 30.4)	3.22 (3.09, 3.35)	1.00 (0.92, 1.09)
≥ 10	236	197.1 (190.1, 204.2)	7.10 (7.02, 7.17)	4.45 (4.41, 4.50)	63.1 (59.4, 66.8)	25.7 (23.3, 28.1)	28.7 (25.3, 32.5)	3.46 (3.34, 3.58) *	1.08 (1.00, 1.16)
*p for trend*	494	*0.725*	*0.616*	*0.807*	*0.057*	*0.918*	*0.289*	*0.001*	*0.111*
**4) Lifetime amount of alcohol (x10**^**3**^**g)**									
Never	173	200.1 (191.8, 208.4)	7.13 (7.04, 7.22)	4.47 (4.42, 4.52)	68.7 (64.3, 73.0)	25.5 (22.7, 28.3)	28.4 (24.4, 32.5)	3.20 (3.06, 3.34)	1.00 (0.90, 1.09)
Mild (<100 x10^3^ g for male, <35 x10^3^ g for female)	185	193.0 (185.4, 200.6)	7.10 (7.02, 7.18)	4.56 (4.41, 4.50)	64.3 (60.3, 68.3) *	24.5 (22.0, 27.1)	27.5 (23.8, 31.2)	3.24 (3.11, 3.37)	1.07 (0.99, 1.09)
Moderate (100–200 x10^3^ g for male, 35–70 x10^3^ g for female)	40	203.6 (191.4, 215.8)	7.00 (6.87, 7.13)	4.44 (4.36, 4.51)	59.9 (53.5, 66.2) *	23.6 (19.5, 27.7)	23.4 (17.4, 29.3)	3.35 (3.14, 3.56)	0.97 (0.83, 1.11)
Heavy (≥200 x10^3^ g for male, ≥70 x10^3^ g for female)	96	195.4 (186.5, 204.3)	7.12 (7.02, 7.21)	4.44 (4.38, 4.50)	62.6 (58.0, 67.3) *	27.7 (24.7, 30.7)	38.8 (24.5, 33.1)	3.68 (3.53, 3.83) *	1.08 (0.98, 1.18)
*p for trend*	494	*0.679*	*0.618*	*0.329*	*0.026*	*0.237*	*0.929*	*<0.001*	*0.316*

Abbreviation:AST, aspartate aminotransferase; ALT, alanine aminotransferase; Ln(GGT), natural logarithmic scale of gamma-glutamyltransferase.

^a^ Data are presented as mean (95% C.I.). Estimated mean and 95% CI were calculated by using multivariable linear regression model adjusted for age, gender, body mass index, regular medication, daily coffee drinking (none, < 1 cup, 1–2 cups, ≥ 3 cups) and smoking (none, < 0.5 pack, 0.5-1 pack, > 1 packs) amounts.

*P-value <0.05 compared to none or never drinkers.

## Discussion

In the present study, strong correlations existed among coffee consumption, alcohol drinking and smoking habits. The associations between those lifestyle habits and LFTs were investigated using multivariable analyses: coffee drinking was significantly associated with lower levels of serum total protein, albumin, and AST, but did not affect TC, ALT, GGT and TB. In addition, cigarette smoking diminished serum total protein and albumin, whereas it raised GGT levels independently. Alcohol drinking independently raised GGT levels. As our best knowledge, this is the first study that demonstrated independent effects of coffee drinking, smoking and alcohol consumption on the comprehensive LFTs commonly used in humans. Several previous studies included only limited test items in LFT and their associations with each of the variables (alcohol, smoking or coffee drinking), but not with all of these common lifestyle habits were reported.

We demonstrated that total protein and albumin levels were decreased by both coffee drinking and smoking. The decrease of serum protein and albumin levels according to increase of daily coffee consumption amount was also confirmed in subjects who had never drunken alcohol nor smoked in the present study (data not shown). Although most previous studies had not mentioned total protein or albumin levels, a study documented that current or past coffee consumption and smoking lower serum albumin, globulin, and all other protein fractions [15]. Moreover, in the chronic hepatitis patients, current smokers were more likely to have lower albumin levels than nonsmokers [16]. However, the biological mechanisms leading to decreased levels of serum protein and albumin by coffee drinking and smoking have not been studied, yet.

Previous epidemiological studies suggested a hepatoprotective effect of coffee drinking on liver function [17]. Furthermore, the protective effects of coffee consumption on AST and ALT have been reported especially in heavy alcohol drinkers [8]. In contrast, several prospective experimental studies demonstrated rather elevated AST and ALT levels after administration of coffee or its ingredient (cafestol) in human subjects, as well as in animal studies [18]. In our study, the heavy alcohol drinkers were only 9.3% of the total subjects, and we could not ascertain the protective effect of coffee on the AST or ALT changes related with alcohol drinking. Although GGT is a sensitive indicator of liver disease, its specificity is not high enough, since numerous environmental factors and drugs can elevate GGT. Coffee has been implicated to reduce GGT concentration because its anti-oxidant ingredients may preserve intracellular homeostasis, which need GGT [19]. However, in our study, GGT level was not independently associated with coffee consumption after adjustment of age, gender, BMI, alcohol and smoking. Alcohol drinking [3] and smoking [20] were well known factors to raise GGT levels. Although BMI is one of the most important factors resulting in GGT elevation [1], several previous studies that have asserted the protective effect of coffee on the GGT did not consider BMI in their estimations [21]. Therefore, such confounding factors could provide a false favorable effect of coffee consumption on the serum GGT concentration.

The present study is the first to describe a detailed effect of smoking on the individual test of LFTs. Previous studies showed that current smokers revealed high GGT compared to non-smokers [22] and people with high GGT levels smoked more [23], which was compatible with our results (Table 5). It was controversial whether smoking could affect aminotransferase activities. Some investigators claimed ALT was increased by smoking [24], while recent studies argued that smoking did not influence AST or ALT, but GGT [25] as our results support. Although our multivariable results showed elevated ALP levels in the current smoker compared to never having smoked or past-smokers, it was not confirmed in the daily or lifetime smoking amounts. Several studies concerning osteoporosis have documented increased serum ALP levels in current smokers, as a marker of bone turnover [26]. Therefore, the effects of smoking on ALP level may be complicated with many extrahepatic mechanisms that did not show a consistent association with smoking in our study after adjustment of many factors.

It was interesting to observe the strong correlations between coffee consumption, alcohol drinking, and smoking habits in our study subjects. In addition, these correlations have been documented in most prior studies [27] suggesting that coffee drinkers are more likely to be alcohol drinkers or cigarette smokers. Therefore, the complete information about these habits is warranted to analyze or interpret reasonably the effect of each habit on LFTs.

In the present study, we carried out a survey on health-check examinee volunteers who were eager to be evaluated about their lifestyle and the effects on their health status, and would provide almost complete responses about alcohol drinking, smoking and coffee consumption; this provided the opportunity to perform a comprehensive analysis of the relationship of these behaviors with LFTs. The strengths of our study included an intensive data collection, and we distinguished participants who had quitted coffee, alcohol and smoking (past users) from those with no history and current users, which made it possible to gain information about lifetime consumption amounts. An additional strength was that we presented independent effects of coffee consumption, smoking, and alcohol drinking on the most commonly used comprehensive items of LFTs, adjusted by extensive confounding factors that included age, gender, BMI, and regular medications. Nevertheless, the lack of evidence of the causal-relationship between lifestyle and LFT changes remained a limitation of our study due to its cross-sectional design. In addition, some heavy alcohol drinker or smoker might be excluded from the present study since 45% of eligible patients refused to respond to the questionnaire. Moreover, coffee-consumption patterns, such as high frequency of instant coffee consumption in Korea, might be a barrier to generalize our results. As we mentioned above, 71% of participants usually consumed instant coffee (57.8%, instant coffee only; 13.2%, instant and brewed coffee, Table 1). Most instant coffee on the market in Korea contains cream and sugar, which is different from brewed coffee that is the primary type of coffee consumed in Western countries [28]. Information about decaffeinated or caffeinated coffee consumption was not recorded because decaffeinated coffee was rarely consumed in Korea.

## Conclusions

High coffee consumption and heavy smoking were both associated with low total protein and albumin levels. High coffee consumption lowered serum AST levels, heavy smoking raised GGT levels, and heavy alcohol drinking raised GGT levels, independently. Because smoking, coffee and alcohol drinking habits showed strong interactions among each other, the association of those habits and LFTs should be carefully analyzed and interpreted. Further studies on the mechanisms of these associations are warranted.

## Abbreviations

LFT: Liver function test; TC: Total cholesterol; ALP: Alkaline phosphatase; TB: Total bilirubin; AST: Aspartate aminotransferase; ALT: Alanine aminotransferase; GGT: γ-glutamyl transferase; BMI: Body mass index; HCC: Hepatocellular carcinoma; HBsAg: Hepatitis B virus surface antigen; HBsAb: Hepatitis B virus surface antibody; anti-HCV: Anti-hepatitis C virus antibody; ANOVA: Analysis of variance; CI: Confidence interval.

## Competing interests

The authors have no conflict of interest to declare.

## Authors’ contributions

ESJ carried out the statistical analysis and drafted the manuscript. S-HJ participated in the study design and coordination and helped to draft the manuscript. SHH and HYK contributed the acquisition of clinical data and the recruitment of subjects. SYA and JL helped the statistical analysis. S-HL, YSP, JHH, J-WK, NK, and DHL contributed the acquisition of data and revised manuscript critically for important intellectual content. All authors read and approved the final manuscript.

## Pre-publication history

The pre-publication history for this paper can be accessed here:

http://www.biomedcentral.com/1471-230X/12/145/prepub

## Supplementary Material

Additional file 1 Personal habits questionnaire form about coffee, alcohol drinking and smoking. Click here for file
