# Supplementary material for: Effects of coffee, smoking, and alcohol on liver function tests: a comprehensive cross-sectional study
Source: BMC Gastroenterol. 2012 Oct 18;12:145. doi: 10.1186/1471-230X-12-145 (PMC3531257; doi:10.1186/1471-230X-12-145)
Supplement: Additional file 1 — Personal habits questionnaire form about coffee, alcohol drinking and smoking. [file 1471-230X-12-145-S1.doc]

**This questionnaire will provide valuable information regarding your experience with alcohol, coffee and cigarettes. In order to best address your specific treatment needs, please fill out this form as completely as possible.**

**1. This section will ask you about your experience with alcohol.**

**1-1) Have you regularly drunk alcohol?**

**□ No → Skip to the next coffee section.**

**□ Yes, but I don’t drink now. (Stopped drinking in year ____)**

**□ Yes, and I still drink.**

**1-2) How many years have you been drinking so far? ( ) years**

**1-3) Think about your past and present drinking experiences and indicate kinds of alcohol, the number of times, and the amount of alcohol consumed at once.**

| **Type of alcoholic beverage** | **Average amount during last year** | | | | | | | | **Average amount of alcohol consumed at once** |
| --- | --- | --- | --- | --- | --- | --- | --- | --- | --- |
|  | **Don’t drink** | **Once a month** | **2-3 times a month** | **Once a week** | **2-3 times a week** | **4-6 times a week** | **Once a day** | **Two or more times a day** |  |
| **Makgulli, other Korean traditional liquor** | **□** | **□** | **□** | **□** | **□** | **□** | **□** | **□** | **( )cc, ( ) glasses** |
| **Wine** | **□** | **□** | **□** | **□** | **□** | **□** | **□** | **□** | **( )cc, ( ) glasses** |
| **Soju** | **□** | **□** | **□** | **□** | **□** | **□** | **□** | **□** | **( )cc, ( ) glasses** |
| **Beer** | **□** | **□** | **□** | **□** | **□** | **□** | **□** | **□** | **( )cc, ( ) glasses** |
| **Whiskey** | **□** | **□** | **□** | **□** | **□** | **□** | **□** | **□** | **( )cc, ( ) glasses** |

**2. The following questions below will ask you about your experience with coffee.**

**2-1) Do you regularly drink coffee?**

**□ No, I hardly drink coffee → Skip to the next smoking section.**

**□ Yes, I don’t drink now. (Stopped drinking in year ____)**

**□ Yes, and I still drink.**

**2-2) How many years have you been drinking coffee so far? ( ) years**

**2-3) What kind of coffee do you usually drink?**

**□ I usually drink instant coffee (vending machine coffee, coffee mix).**

**□ I usually drink brewed coffee.**

**□ I drink both instant and brewed coffee.**

**□ I drink other type of coffee. ( )**

**2-4) How do you drink your coffee?**

**□ With cream and milk**

**□ Black**

**2-5) How often do you drink coffee?**

**□ Once a day or more. → Skip to 2-5)**

**□ 4-6 cups a week**

**□ 2-3 cups a week.**

**□ Once a week**

**□ 2-3 times a month**

**□ Once a month → Skip to the next smoking section.**

**2-6) If you drink coffee everyday, how many cups do you drink a day?**

**□ 1-2 cups □ 3-4 cups □ 5-6 cups □ more than 7 cups**

**3. The following questions will ask you about your experience with smoking.**

**3-1) Have you ever smoked cigarettes?**

**□ No → End of Questionnaire**

**□ Yes, but I don’t smoke now. (has been ____yrs. since I stopped)**

**□ Yes, and I still smoke.**

**3-2) At what age did you start smoking?**

**□ ( ) years old □ Don’t know.**

**3-3) How many years have you been smoking?**

**□ ( ) years □ Don’t know.**

**3-4) How many cigarettes on average did you smoke a day?**

**□ Average ( ) cigarettes**

**3-5) Have you lived with a person who smoked more than 20 packs of cigarettes?**

**□ No □ Yes □ Don’t know.**
